# Report of the Veterinary Department of the Minnesota State Board of Health

**Published:** 1898-03

**Authors:** 


### REPORT OF THE VETERINARY DEPARTMENT OF THE MINNESOTA STATE BOARD OF HEALTH FOR QUARTER AND YEAR ENDING JANUARY 1, 1898.

MR. PRESIDENT AND GENTLEMEN OF THE BOARD: I take pleasure in presenting the following report of work done in the veterinary department during the quarter and year ending January 1, 1898.

The work during the entire year has been largely with glanders and hog-cholera, although there have been scattered outbreaks of other diseases, especially tuberculosis, black-leg, rabies, and corn-stalk-disease among cattle. The latter has been unusually prevalent during this seaaon, and is now reported, I believe, for the first time, to this board.

### HOG-CHOLERA.

*Methods adopted during the present season.* I believe that the most-lasting and important benefit that will result from our hog-cholera work of 1897 will be the good that came from the campaign of education. I refer to the printed matter which has been distributed freely throughout the State; to the assistance given us by the local newspapers, and to the work of Secretary Bracken, who has met a large number of farmers and local health officers at various public meetings throughout the infected portion of the

State. Mr. Kenning has done similar work in Renville and McLeod Counties. In addition to the printed matter and newspaper work, I have been able, by the courtesy of Superintendent Gregg, to present the subject of hog-cholera to many large audiences of farmers.

I have felt throughout the season that in thus getting the owners and local health-officers, including town-boards, ready for next season's work something material was being accomplished, although the work this year has not been as satisfactory as we hoped at the beginning it might be. We have endeavored to treat hog-cholera just as the sanitarian in the field of human medicine deals with smallpox or yellow-fever—*i. e.*, by individual and local quarantine.

The following stock letter (*d*) and circular of instruction concerning hog-cholera illustrate the instruction which local health-officers have received for their work.

At a previous meeting of the Board, on August 10th, a series of rules were adopted for the control of hog-cholera in Minnesota, and put in force August 20th. These rules divided the State into two districts, A and B; A being declared a generally infected district, and certain quarantine regulations were established to control the traffic within each district, from one district to the other, and from other States into these districts. A copy of these rules is attached hereto.

(*d*) *Rules for Controlling Hog-cholera in Minnesota.*

These rules do not interfere with any shipments of swine, for slaughter, into any stock-yards of the State that may be located within district "A," and these rules are so framed that the large markets of the State are located within district "A."

1. The following counties were more or less generally infected with hog-cholera during 1896, and 1897 up to date, and are hereby declared an infected district, and designated as district "A." Counties: Brown, Watonwan, Martin, Freeborn, Faribault, Blue Earth, Nicollet, Le Sueur, Waseca, Hennepin, and Ramsey, excepting the State fair-grounds. This district is subject to such modifications as the State Board of Health may see fit to make from time to time.

2. All that portion of the State not included in district "A" shall be known as district "B."

3. Shipment of swine from any point in district "A" to be unloaded in district "B," and all other movements of swine, whether they are driven on foot or hauled in wagons, from any point in district "A" into district "B," are hereby prohibited.

4. All shipments from points outside of this State, to be unloaded within this State, are prohibited, except as provided in rule 5.

5. Hogs shipped from any other State into Minnesota must be crated, shipped in other than stock-cars, and accompanied by a certificate signed by a veterinarian or physician that they are free from the disease when shipped and come from an uninfected district.

6. Hogs shipped from point to point in district "B" must be crated and shipped in other than stock-cars.

7. All outbreaks of suspected hog-cholera in district "B" and in such places as may be deemed practical in district "A" shall be rigidly quarantined.

8. Shippers of hogs from point to point in district "A" shall be required by the railroad agent to sign a statement to the effect that such hogs are for slaughter only and within five days after reaching destination.

Railroad agents should preserve all certificates demanded by these rules for the protection of their companies in case any shipment of hogs should be followed by an outbreak of disease.

9. All exhibitions of swine at county fairs in district "A" are hereby forbidden.

10. All cars in which hogs are shipped into or through this State shall be constructed so as to prevent the escape of manure and litter.

These rules shall go into effect August 20, 1897, and continue in force until altered or annulled by the State Board of Health.

"Any person violating any rule or regulation made by the State Board of Health, or any order made by such board under the authority hereof, shall be guilty of a misdemeanor and be punished by a fine of not less than twenty-five (25) or more than one hundred (100) dollars, or by imprisonment for not less than thirty (30) or more than ninety (90) days. Complaints for violating the provisions of this act, or for violating any rule or regulation made by any board of health under its authority, may be made by any member or authorized agent of any such board, or by any citizen of this State."—From Section 9, Chapter 233, Laws of 1897.

H. M. Bracken, M.D.,
Secretary and Executive Officer.

*Quarantine line.* In pursuance of a suggestion offered by Hon. John Cooper, of St. Cloud, we have attempted to establish a quarantine-line across the State on a line with McLeod and Renville Counties, this being north of the generally infected district. Mr. Kenning has recently completed a second trip over these two counties for the purpose of estimating the results of his first trip. It is evident that we can accomplish considerable good in that way.

*Government experiment in Iowa.* As you are doubtless aware, the Government has been carrying on an interesting experiment in Page County, Iowa, the results of which may be of considerable value, and may possibly modify our method in this State. The Government has been trying the experiment in that county of controlling hog-cholera by purchase and slaughter of infected herds and by serum-vaccination. The results have not been published as yet. I am awaiting this report with great interest.

*General estimate of results.* A careful study of the work that has been in operation during the past season, as shown by our statistics and partially illustrated by the map which I show to-day, are somewhat disappointing, and yet I feel that we have accomplished something for Minnesota stock-interests. I see now that at the beginning of the work in 1897 I hoped for far more than was possible. In view of the fact that when the work was undertaken at least thirty-nine counties were affected, the present situation is not discouraging. (Maps shown and explained.)

In order to study our results fairly, we must divide the State into two districts:

1. In previously and badly affected districts our results were unsatisfactory; but we have been able, among other things, to render less rapid the spread of disease, as shown by the list of townships and counties affected, and this is also true of the territory immediately bordering on the generally infected district. This district includes the following counties: Rock, Nobles, Jackson, Martin, Faribault, Freeborn, Fillmore, Waseca, Blue Earth, Watonwan, Cottonwood, Murray, Nicollet, Le Sueur, and McLeod.

2. In portions of the State that were infected during 1897 for the first time our efforts have been much more satisfactory. In many cases the disease did not spread beyond the township in which it first appeared. There are forty townships, indicated by the large red spots on this map, which were infected at some time between January 1st and November 1st, but the disease had entirely disappeared before November 1st. So far as I have been able to obtain reports from the chairman of the various townships, there have been but two townships in seven counties infected since November 1st, for the first time.

The total number of counties infected in 1896 were thirty-nine; total number counties infected in 1897 were thirty-nine; number new counties infected in 1897, nine; number infected in 1896, but not in 1897, nine. In comparing these figures we must bear in mind that we have very accurate reports both for townships and counties, location of outbreaks, financial losses, etc., for 1897, and inaccurate reports for 1896. It is quite probable that if we could have collected information in the same way in 1896, that the number of counties and total loss for 1896 would have been much larger. In the map shown the blue crosses indicate the counties infected in 1896.

*Experiments with hog-cholera serum.* I have been disappointed with the results of our hog-cholera serum experiments. The beginning of this experiment and the early portion of our work was reported at a previous meeting of the board. As you will remember, I announced at that time that I had made arrangements with Dr. A. T. Peters, of the Nebraska Experiment Station, to unite in our work, so far as this matter was concerned, during the present season. Dr. Peters was to do the laboratory work and furnish the serum, and the veterinary department of the State Board of Health, in connection with the veterinary division of the Experiment Station, to plan and execute the field experiments. Our plan for field experiments was as follows:

*Plan for Field Experiments with Serum in Outbreaks of Hog-cholera.*

1. Protection: *a.* Original prevention, that is, of swine that have not been exposed. Select a small herd of well animals that, so far as known, have never been exposed to the infection, and inoculate one-half of them. After ten days send out inoculated pigs with others that have not been inoculated for controls, to be placed in herds where the disease is present.

*b.* Exposed and possibly already infected swine. Select a herd in which undoubted hog-cholera has appeared, and in which the entire herd has been exposed to infection; inoculate one-half of the herd, using a separate earmark for those that were sick and those that were well when inoculated, and keep complete records. (Odd numbers "inoculated;" even numbers "not inoculated." In right ear for "sick," and left ear for "well." Keep careful records of number and ear of those that die.)

2. Experiments as to curative value: Carefully kept records of hogs that were sick when inoculated, when compared with the records of other sick hogs that were not inoculated, will give a very fair estimate of the therapeutic value of the serum.

Doses: 10 to 50 lbs., 2.5 c.c.; 50 to 100 lbs., 5 c.c.; 100 to 200 lbs., 10 c.c. 10 c.c. of three days' old culture hog-cholera bacilli to be added to each 100 c.c. of serum, then well stirred.

*Results.* Our results, briefly summarized, are as follows:

Under the heading of "Original Prevention," the Experiment Station purchased ten shoats, weighing about seventy-five pounds each. We vaccinated half of them, and all were given metal ear-tags according to a system of ear-tagging. After ten days these shoats were sent out in pairs, one vaccinated with one control, and exposed in pens and small yards to undoubted hog-cholera. One pair was sent to each of the following places: New Ulm, Bird Island, Sauk Centre, Owatonna, and Willmar.

The results of this experiment demonstrated that the vaccinated pigs had developed no immunity, as nine of the pigs have died, and in many cases the vaccinated one died sooner than the other.

In our experiment with "exposed and possibly already infected swine," a total number of 236 hogs were used on twelve different farms and in different portions of the State. Of 119 vaccinated hogs, 32 died; of 117 not vaccinated, 27 died.

It should be explained that this was probably not a fair test of Dr. Peters' serum, because of unfavorable conditions of shipment and pure culture inoculation of the serum.

While our results have been very unsatisfactory they have not been discouraging, and I hope to continue the work under more favorable conditions during 1898, and if necessary for many years to come. I am satisfied that a most important factor in the solution of the hog-cholera problem lies within this matter of a vaccine that shall be preventive and possibly curative.

I wish to submit the following proposed circulars of information concerning hog-cholera. Many of the most important points in these hog-cholera circulars were suggested by Dr. Bracken.

*Circular of Information for Local Health-Officer—For Quarantining Hogs Suffering from any Suspicious Disease.*

The purpose of the Board of Health in quarantining any suspicious disease among hogs is to protect the financial interests of the hog-raisers. The proper quarantine of hogs imposes no hardship upon either the owner or the hogs themselves. Valuable time is often wasted before quarantine is established, and this permits serious spread of the disease to take place, making its control much more difficult.

In all cases where hogs begin to sicken and die during the prevalence of hog-cholera the disease should be reported to the local health-officer or chairman of the Town Board. Quarantine should be established at once, for it is a simple matter to release quarantine, and should it be proved that the disease is not hog-cholera or swine-plague, no harm has been done by such quarantine. All health-officers and acting health-officers are therefore instructed to see that all suspicious outbreaks of disease among hogs are properly quarantined.

The first step in quarantining a herd of hogs has been accomplished when the proper placard has been dated and signed by the proper officer and posted in a conspicuous place upon or near the yards, pens, or sheds in which the hogs are confined. The health-officer should then explain to the owner or keeper the nature and conditions of quarantine, and thereafter see that the conditions are rigidly enforced until quarantine is released.

Farmers should not only be permitted, but urged to dispose of marketable hogs for slaughter as soon as suspected hog-cholera appears in a neighborhood. Hogs from an infected neighborhood may be shipped for slaughter to any place where inspection is conducted by Government, State, or local health-officers, except as otherwise provided by the State Board of Health.

It is sometimes advisable at the beginning of an outbreak to kill and bury, or burn, the hogs as fast as they show the first suspicious symptoms of the disease.

If hogs are confined in small pens or yards after the disease has appeared, such pens or yards should be kept as clean as possible by removing the manure frequently and keeping it in a compact pile, making alternate layers of manure and lime, for the manure is very infectious.

The troughs should be scalded once daily with boiling water. Little bedding should be allowed, and that should be frequently removed and burned. The floors may be disinfected by a 2 per cent. solution of crude carbolic acid in water; lime should be sprinkled freely over the yard.

If swill-barrel or tank is used, it should be emptied twice a week, and scalded with boiling water, and then exposed to the sun until thoroughly dry.

If the pens or small yards can be kept thoroughly clean, as herein suggested, it is better to keep the hogs in such places than in the larger pastures and yards; otherwise it is better to give them a larger range.

The disease now prevailing in different portions of the State varies in symptoms in different localities and in different herds. It may be set down as a rule, however, that when any infectious (catching) disease appears among swine it is probably hog-cholera, modified more or less in symptoms by certain well-known complications.

*Circular of Information for Shipment of Hogs from an Infected District.*

The local health-officer must insist, whenever it is practicable, that hogs which are removed from an infected farm before the quarantine card is posted, must be hauled in wagons and loaded directly from them into the cars.

Racks and wagon-boxes used for hauling such hogs must be tight, and so constructed at the bottom as to prevent the scattering of manure and litter along the highway.

Racks or wagon-boxes which have been used for transporting such hogs must be thoroughly disinfected as soon as the work is finished. All parts of the wagon that have come in contact with the hogs or litter must be thoroughly saturated with the disinfecting fluid. Two per cent. solution of crude carbolic acid in water is cheap and effective.

Neighbors on whose farms the disease has not yet appeared should never be allowed to help haul the hogs from an infected farm, as there is always great danger that the disease will be spread from farm to farm by such action.

In response to a suggestion at the last meeting, we have issued the following circular on "So-called Hog-cholera Cures."

This has been sent to nearly all the papers in the infected portion of the State, and sent out freely to chairmen of town boards and other local health-officers. This has brought us into some conflict with hog-cholera-cure firms, but no serious trouble.

*Concerning so-called Hog-cholera Cures.*

At a meeting of the State Board of Health, October 12, 1897, it was decided that the veterinary department should issue a circular of advice to farmers and stockmen concerning the many so-called cures for hog-cholera and the men who are going over the State selling them. These men are teaching that the disease is not contagious, and that it can be cured by their special preparation, that all other medicines are useless, etc. Some are even teaching the silly doctrine that hog-cholera and swine-plague are caused by certain methods or systems of feeding.

Hog-cholera has been proved infectious by many different men and by many careful experiments. Practical experience supports these scientific experiments, and so it is difficult to understand how anyone who has had experience with the disease can think otherwise than that it is infectious.

The hog-cholera-cure agents are themselves a constant source of danger, as they go from farm to farm. Owners and local health-officers should not allow them to go near the hogs upon any farm where the disease has not appeared, and they violate the law when they go into pens or yards where hogs have been quarantined by local or State Board of Health.

From the very nature of the disease it seems impossible for drugs or patent medicines of any kind to have any curative value, and money paid for drugs or chemicals to be used as cures is therefore wasted. Such drugs as crude carbolic acid, corrosive sublimate, creolin, lime, etc., have value of disinfectants only.

It is unfortunate that farmers should believe in these so-called cures, for, as a consequence, they usually grow very careless about matters of quarantine and protection from infection.

Editors will do a good thing for their farmer-readers if they repeatedly urge them to spend no money for any hog-cholera cures.

Mr. John Cownie, Vice-President of the State Agricultural Society of Iowa, has recently superintended a test of one of the most prominent of these hog-cholera cures, and his conclusions, as published at length in Wallace's *Farmer and Dairyman*, of October 15th, are as follows: "So far the disease which is now destroying the swine herds of our State has baffled all efforts to cure or even to control it, and each and every one of the so-called 'hog-cholera cures' now on the market have proved, when put to a fair and honest test in a herd really affected by the disease, to be without merit and absolutely worthless as a cure for this dread complaint." Henry Wallace, well-known all over the West as one of the most prominent agricultural editors, followed this experiment closely from beginning to end, and certifies to the correctness of Mr. Cownie's statement.

The only prospect now in sight for the cure or medical prevention of this disease seems to lie in the way of blood-serum or antitoxin. We hope it will not be many years before some vaccine will be discovered which will be as sure a preventive of hog-cholera as vaccination is for smallpox, or as good a treatment as antitoxin for diphtheria; but these things are all in the experimental stage as yet, and it may be a long time before any can be positively recommended. Meanwhile the farmers of Minnesota should not add to their losses by spending money for worthless hog-cholera medicines. They should, however, do everything in their power to prevent the spread of the disease by excluding the common carriers of infection from their herds of hogs.

M. H. Reynolds, M.D., V.M.,
Director of the Veterinary Department of the State Board of Health.

*Newspapers.* I think that this board should recognize in some public way the generous assistance that has been given us by the newspapers of the State. They have printed very freely the large amount of matter which has been sent them concerning hog-cholera, and in this way have benefited their farmer-readers and greatly assisted us in the work.

*Plans for* 1898. I am anxious to learn the results of the Government experiment in Page County, Iowa, before submitting definite plans for dealing with hog-cholera in 1898, and had hoped to receive a full report before this report was submitted.

It will evidently be necessary to alter the rules for the control of hog-cholera in Minnesota as adopted by this Board on August 10, 1897. The rules must be materially modified, certain ones abolished, new ones added, and others modified. I would suggest that this matter be referred to the Committee on Infectious Diseases of Animals and the Executive Committee, with power to act jointly.

I wish to continue my work with preventive and possibly curative vaccination, as suggested elsewhere in this report.

(To be continued.)

---

A New Antiseptic. A new German antiseptic, called protagol, is a compound of silver and protein. A 1 per cent. solution is reported to destroy the bacteria of anthrax and enteric fever.

